# Supplementary material for: Arabidopsis SBT5.2 and SBT1.7 subtilases mediate C-terminal cleavage of flg22 epitope from bacterial flagellin
Source: Nat Commun. 2024 May 4;15:3762. doi: 10.1038/s41467-024-48108-4 (PMC11069567; doi:10.1038/s41467-024-48108-4)
Supplement: Supplementary file 1 — Supplementary Information [file 41467_2024_48108_MOESM1_ESM.pdf]

Supplementary Information

Arabidopsis SBT5.2 and SBT1.7 subtilases mediate C-terminal cleavage of flg22 epitope from bacterial flagellin

Sayaka Matsui, Saki Noda, Keiko Kuwata, Mika Nomoto, Yasuomi Tada,

Hidefumi Shinohara and Yoshikatsu Matsubayashi*

Nature Communications (2024)

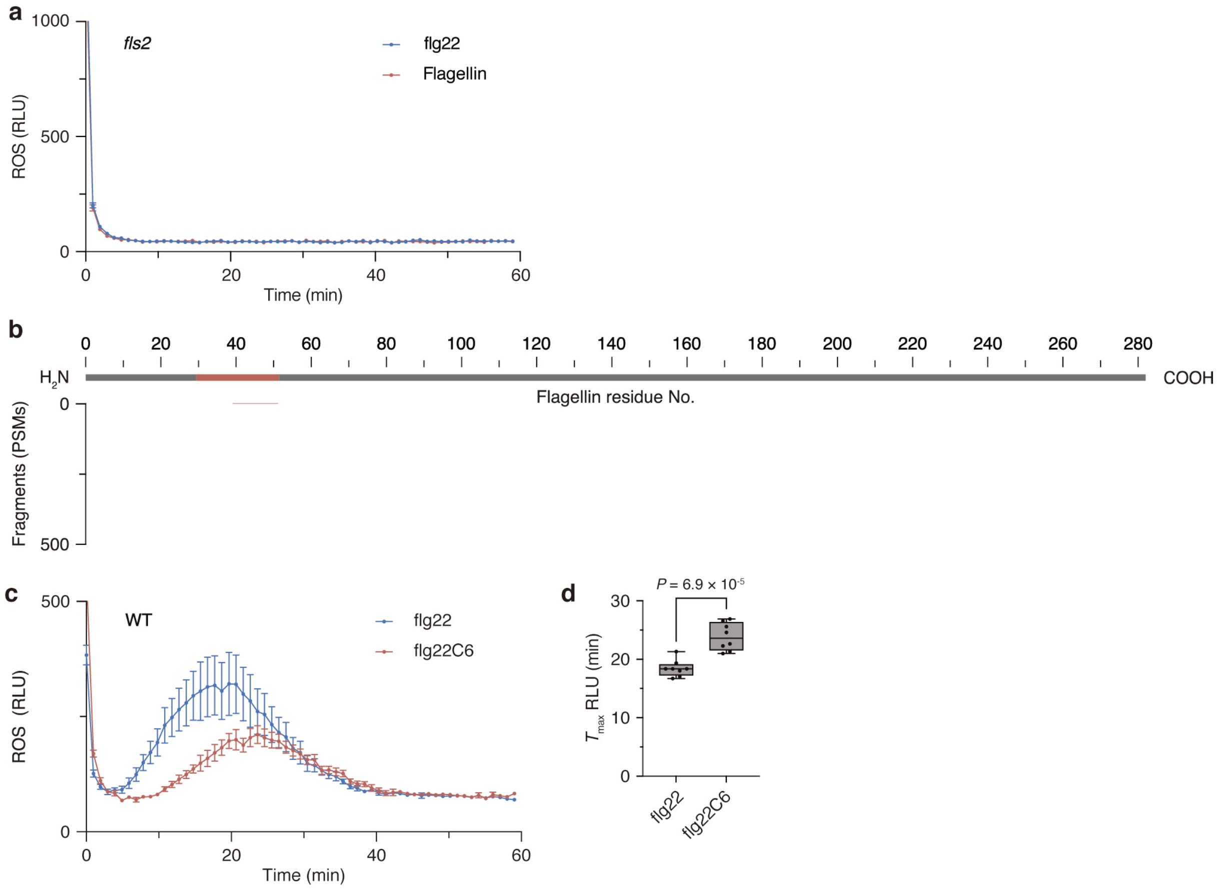

Supplementary Fig. 1. Proteolytic release of immunogenic peptides from flagellin. (a) Absence of ROS burst in *fls2* mutant leaf disks upon elicitor exposure. Leaf disks from *fls2* plants were incubated with 100 nM flg22 peptide or flagellin protein. ROS production was measured every 1 min over 60 min. Values represent the mean ± SE (n = 6 biologically independent samples). (b) Fragmentation pattern of mock-treated flagellin, showing only few fragments. (c) Delayed ROS production upon flg22C6 exposure. Leaf disks from WT plants were incubated with 100 nM flg22 or flg22C6 peptides. ROS production was measured every 1 min over 60 min. Values represent the mean ± SE (n = 8 biologically independent samples). (d) Comparison of time at which the maximum response (T_{max}) was observed. (n = 8 biologically independent samples). Boxes represent 25th to 75th percentile range, the line within the box marks the median and the whiskers represent the minimum and maximum values. *P* values were calculated by two-tailed non-paired Student's *t* test. Source data are provided as a Source Data file.

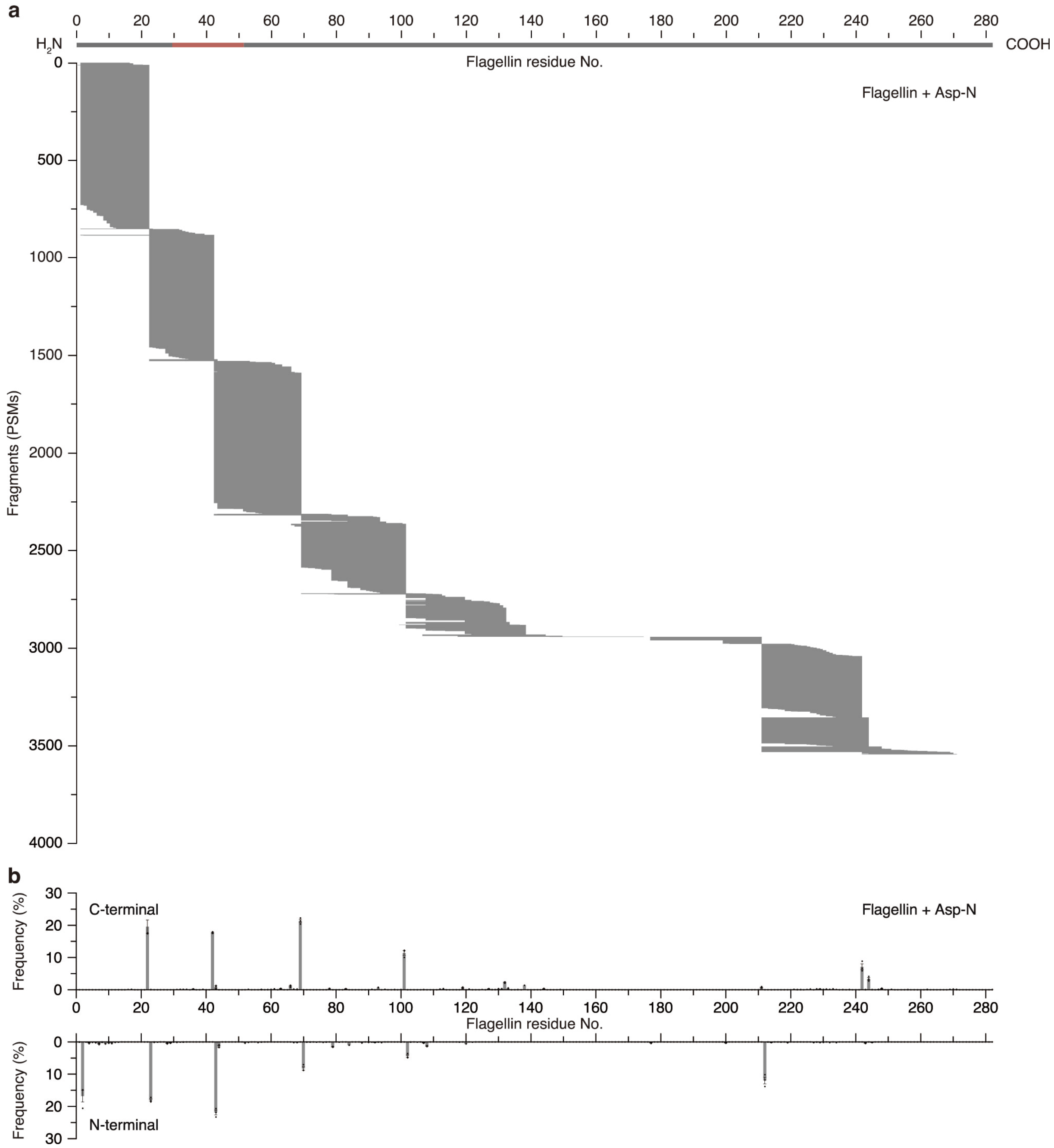

Supplementary Fig. 2. Cleavage patterns of flagellin digested with endoproteinase Asp-N. (a) Representative fragmentation pattern of flagellin digested with endoproteinase Asp-N. Flagellin protein was incubated overnight with endoproteinase Asp-N, and the digested peptide fragments were analyzed by nano-LC-MS/MS. Each PSM was mapped against the amino acid sequence of flagellin. (b) Frequency at which each amino acid residue is the C-terminal end or N-terminal end of the peptides following digestion with Asp-N. Values represent the mean ± SE (n = 3 biologically independent samples). Source data are provided as a Source Data file.

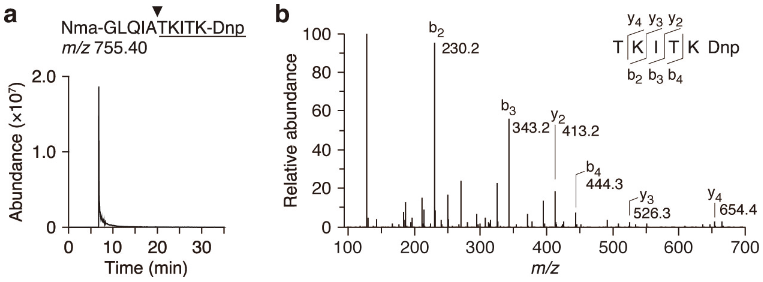

Supplementary Fig. 3. Detection of cleavage activity at the C-terminus of the flg22 epitope within flagellin by fluorescence-quenching substrate. (a) *In vitro* proteolytic assay of Nma-flg[47-56]-Dnp with *Arabidopsis* submerged culture medium. Representative selected ion chromatogram (SIC) is shown for TKITK-Dnp. (b) MS/MS spectrum of the precursor ion at *m*/*z* 755.40 corresponding to TKITK-Dnp.

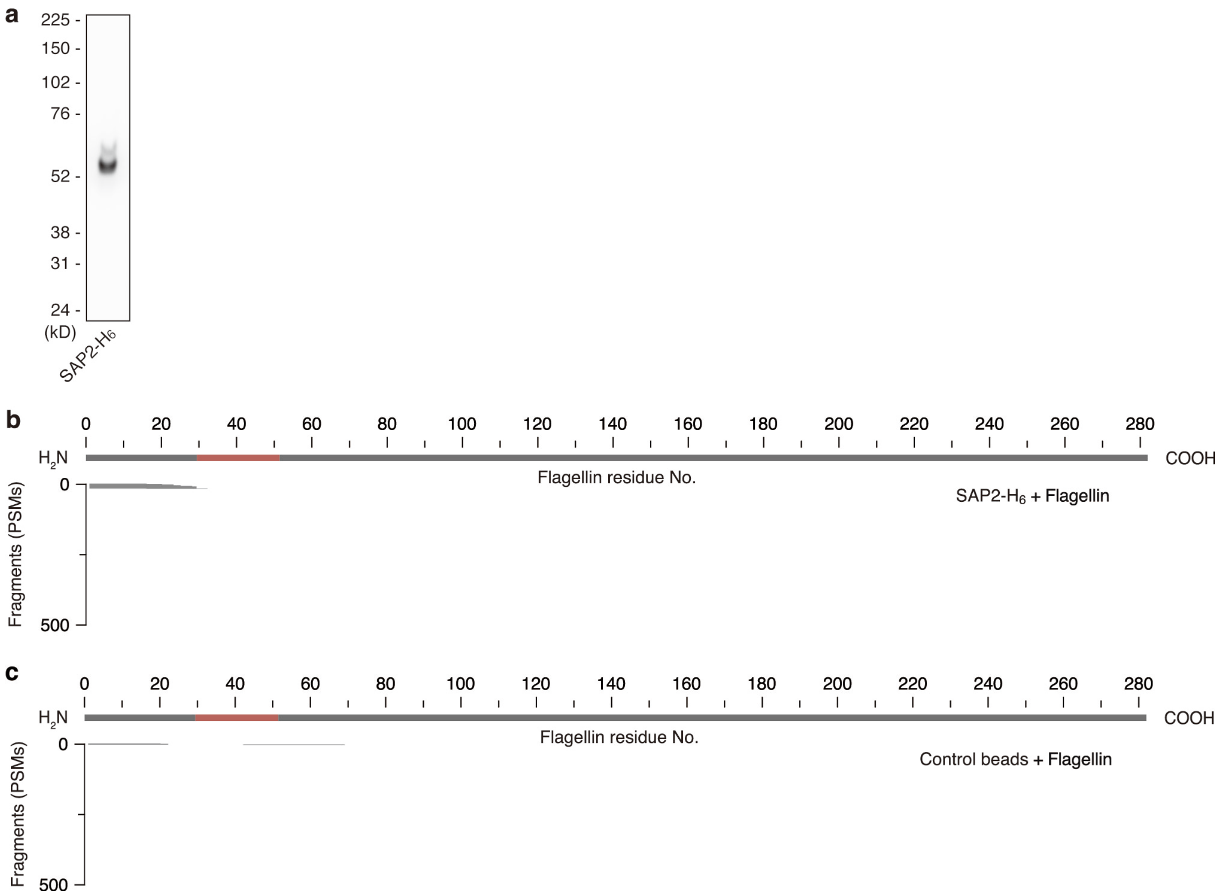

Supplementary Fig. 4. Specificity of cleavage activity by SBT5.2 at the C-terminus of the flg22 epitope. (a) *Agrobacterium*-mediated transient expression of His-tagged SAP2 (SAP2-H_6) in *N. benthamiana*. Apoplast fluids were extracted from agroinfiltrated leaves and subjected to SDS-PAGE followed by western blotting using HRP-conjugated anti-His tag antibody. The experiment was independently repeated three times with similar results. (b) Fragmentation pattern of flagellin digested with SAP2-H_6. Flagellin protein was incubated for 1 h with bead-captured SAP2-H_6, and the resulting peptide fragments were analyzed by nano-LC-MS/MS. (c) Fragmentation pattern of flagellin incubated with control beads, showing only few fragments. Source data are provided as a Source Data file.

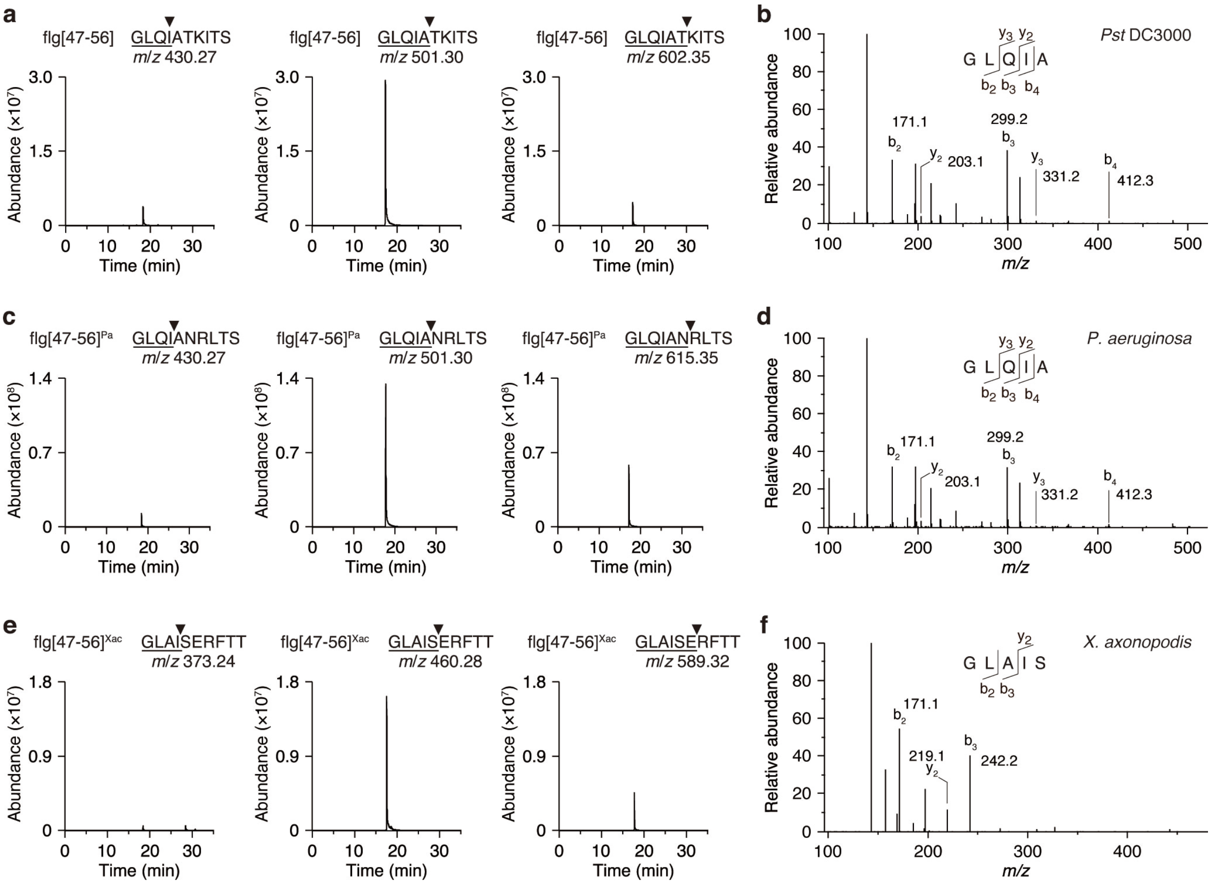

Supplementary Fig. 5. SBT5.2 cleaves the C-terminus of the flg22 domain of flagellins derived from various bacterial species. (a) *In vitro* proteolytic assay of the flg[47-56] peptide derived from *Pst* DC3000 by SBT5.2-H_6. Representative SICs are shown for GLQI, GLQIA, and GLQIAT. (b) MS/MS spectrum of the precursor ion at *m*/*z* 501.30 corresponding to GLQIA. (c) Proteolytic assay of flg[47-56]^{Pa} peptide derived from *Pseudomonas aeruginosa* by SBT5.2-H_6. (d) MS/MS spectrum of the precursor ion at *m*/*z* 501.30 corresponding to GLQIA. (e) Proteolytic assay of flg[47-56]^{Xac} peptide derived from *Xanthomonas axonopodis* pv. *citri* by SBT5.2-H_6. (f) MS/MS spectrum of the precursor ion at *m*/*z* 460.28 corresponding to GLAIS.

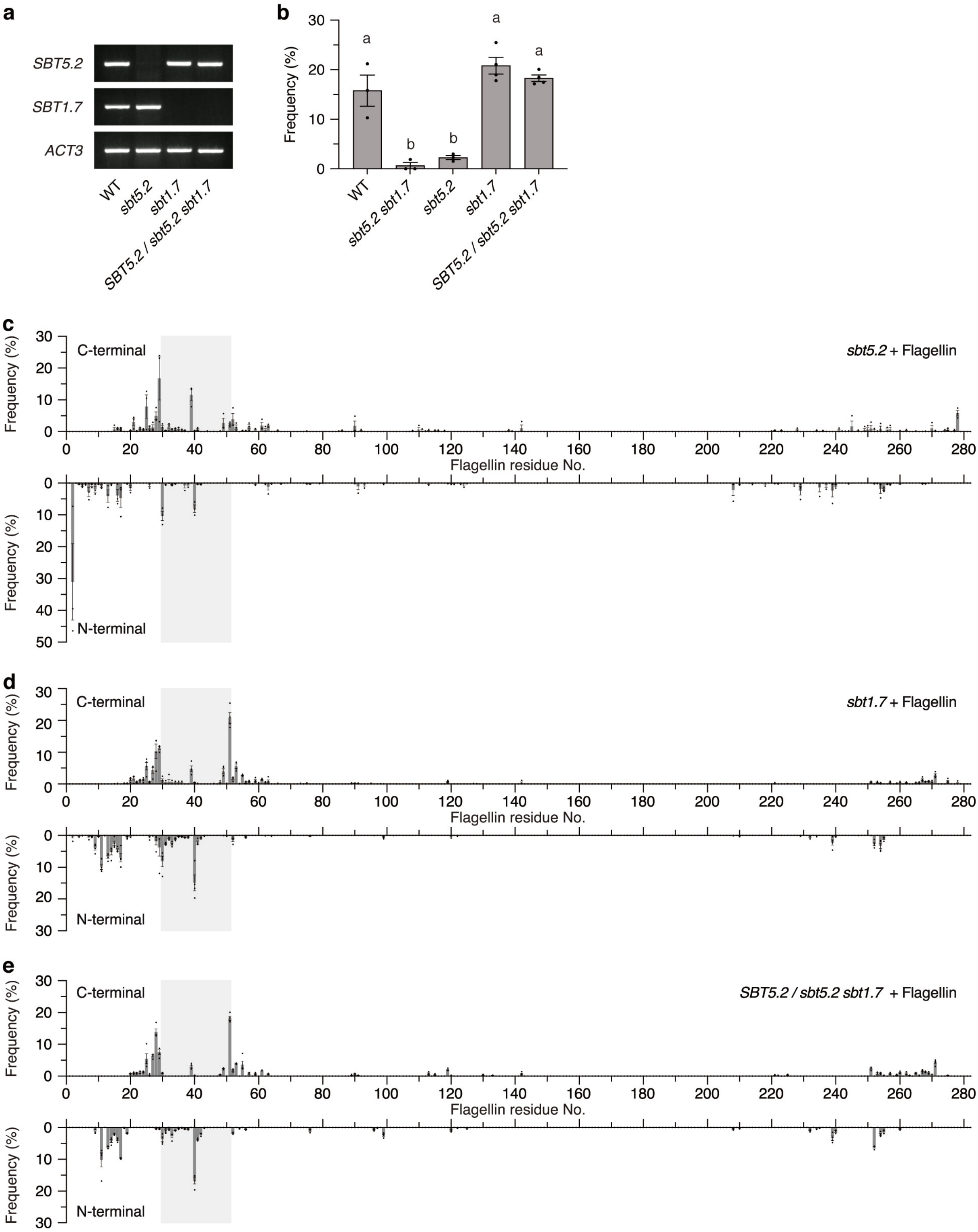

Supplementary Fig. 6. Proteolytic phenotypes of *sbt* mutants and complemented lines. (a) Absence of *SBT5.2* and *SBT1.7* transcripts in the *sbt5.2* and *sbt1.7* single mutants, as verified by RT-PCR. *ACT3* was used as an internal control. (b) Comparison of proteolytic activity at the C-terminus of the flg22 domain (Ala^{51}-Thr^{52}) in submerged culture medium from WT, *sbt5.2* single, *sbt1.7* single and the *sbt5.2 sbt1.7* double-mutant complemented with *SBT5.2* gene. Different letters indicate a significant difference with others (mean ± SE, $P <$

0.05, one-way ANOVA followed by Tukey's test, n = 3 biologically independent samples for WT, *sbt5.2* and *sbt5.2 sbt1.7*, n = 4 biologically independent samples for *sbt1.7* and *SBT5.2 / sbt5.2 sbt1.7*). (c) Fragmentation patterns of flagellin upon incubation in submerged culture medium from *sbt5.2* single mutant. The gray box indicates the flg22 epitope. Values represent the mean ± SE (n = 3 biologically independent samples). (d) Fragmentation patterns of flagellin upon incubation in submerged culture medium from *sbt1.7* single mutant. Values represent the mean ± SE (n = 4 biologically independent samples). (e) Fragmentation patterns of flagellin upon incubation in submerged culture medium of *sbt5.2 sbt1.7* double mutant complemented with *SBT5.2.* Values represent the mean ± SE (n = 4 biologically independent samples). Source data are provided as a Source Data file.
