# Supplementary material for: Arabidopsis SBT5.2 and SBT1.7 subtilases mediate C-terminal cleavage of flg22 epitope from bacterial flagellin
Source: Nat Commun. 2024 May 4;15:3762. doi: 10.1038/s41467-024-48108-4 (PMC11069567; doi:10.1038/s41467-024-48108-4)
Supplement: Supplementary file 3 — Description of additional supplementary files [file 41467_2024_48108_MOESM3_ESM.pdf]

Description of Additional Supplementary Files

Supplementary Data 1. Proteomic comparison of the abundance of secreted proteins in leaf-derived apoplastic fluid and submerged culture medium. Protein abundance was estimated using spectral counts (PSMs).

Supplementary Data 2. Proteins identified in the fluorescence-positive gel fragments and in the fluorescence-negative gel fragment used as a control. Protein abundance was estimated using spectral counts (PSMs).

Supplementary Data 3. Contaminant proteins detected in *Arabidopsis* SBT5.2-H_6 or SBT1.7-H_6 immobilized on Ni-Sepharose beads. Protein abundance was estimated using spectral counts (PSMs).

Supplementary Data 4. Primer sequences used in this study.
