# Supplementary material for: Arabidopsis SBT5.2 and SBT1.7 subtilases mediate C-terminal cleavage of flg22 epitope from bacterial flagellin
Source: Nat Commun. 2024 May 4;15:3762. doi: 10.1038/s41467-024-48108-4 (PMC11069567; doi:10.1038/s41467-024-48108-4)
Supplement: Supplementary file 8 — Reporting Summary [file 41467_2024_48108_MOESM8_ESM.pdf]

Corresponding author(s): Yoshikatsu Matsubayashi

Last updated by author(s): Apr 12, 2024

# Reporting Summary

Nature Portfolio wishes to improve the reproducibility of the work that we publish. This form provides structure for consistency and transparency in reporting. For further information on Nature Portfolio policies, see our Editorial Policies and the Editorial Policy Checklist.

## Statistics

For all statistical analyses, confirm that the following items are present in the figure legend, table legend, main text, or Methods section.

| n/a | Confirmed | |
|---|---|---|
| ☐ | ☒ | The exact sample size (*n*) for each experimental group/condition, given as a discrete number and unit of measurement |
| ☐ | ☒ | A statement on whether measurements were taken from distinct samples or whether the same sample was measured repeatedly |
| ☐ | ☒ | The statistical test(s) used AND whether they are one- or two-sided<br>*Only common tests should be described solely by name; describe more complex techniques in the Methods section.* |
| ☒ | ☐ | A description of all covariates tested |
| ☒ | ☐ | A description of any assumptions or corrections, such as tests of normality and adjustment for multiple comparisons |
| ☐ | ☒ | A full description of the statistical parameters including central tendency (e.g. means) or other basic estimates (e.g. regression coefficient) AND variation (e.g. standard deviation) or associated estimates of uncertainty (e.g. confidence intervals) |
| ☐ | ☒ | For null hypothesis testing, the test statistic (e.g. $F$, $t$, $r$) with confidence intervals, effect sizes, degrees of freedom and $P$ value noted<br>*Give P values as exact values whenever suitable.* |
| ☒ | ☐ | For Bayesian analysis, information on the choice of priors and Markov chain Monte Carlo settings |
| ☒ | ☐ | For hierarchical and complex designs, identification of the appropriate level for tests and full reporting of outcomes |
| ☒ | ☐ | Estimates of effect sizes (e.g. Cohen's *d*, Pearson's *r*), indicating how they were calculated |

*Our web collection on statistics for biologists contains articles on many of the points above.*

## Software and code

Policy information about availability of computer code

| | |
|---|---|
| Data collection | Nano LC-MS/MS analysis: Thermo Xcalibur 3.0.63<br>Nano LC-MS analysis: Thermo LTQ Tune Plus 2.5.5<br>Luminescence/ Fluorescence Intensity Measurements: PerkinElmer VICTOR Nivo control software 4.5.0<br>Western blot detection: GE ImageQuant LAS 4000 mini control software 1.2.0.101 |
| Data analysis | Proteomics analysis: Thermo Scientific Proteome Discoverer 2.3, 2.4, 2.5 , Microsoft Excel 2019, 2021<br>Statistical analysis and graph drawing: GraphPad Prism 9.0 |

For manuscripts utilizing custom algorithms or software that are central to the research but not yet described in published literature, software must be made available to editors and reviewers. We strongly encourage code deposition in a community repository (e.g. GitHub). See the Nature Portfolio guidelines for submitting code & software for further information.

# Data

Policy information about availability of data

All manuscripts must include a data availability statement. This statement should provide the following information, where applicable:

- Accession codes, unique identifiers, or web links for publicly available datasets
- A description of any restrictions on data availability
- For clinical datasets or third party data, please ensure that the statement adheres to our policy

All data supporting the findings of this study are available in the paper and the Supplementary Information. All other data that support the findings of this study are available from the corresponding author upon request. The raw MS data have been deposited in the ProteomeXchange Consortium via PRIDE partner repository as accession number PXD044549. Source data are provided with this paper.

# Research involving human participants, their data, or biological material

Policy information about studies with human participants or human data. See also policy information about sex, gender (identity/presentation), and sexual orientation and race, ethnicity and racism.

| | |
|---|---|
| Reporting on sex and gender | Not applicable to this study |
| Reporting on race, ethnicity, or other socially relevant groupings | Not applicable to this study |
| Population characteristics | Not applicable to this study |
| Recruitment | Not applicable to this study |
| Ethics oversight | Not applicable to this study |

Note that full information on the approval of the study protocol must also be provided in the manuscript.

# Field-specific reporting

Please select the one below that is the best fit for your research. If you are not sure, read the appropriate sections before making your selection.

☒ Life sciences ☐ Behavioural & social sciences ☐ Ecological, evolutionary & environmental sciences

For a reference copy of the document with all sections, see nature.com/documents/nr-reporting-summary-flat.pdf

# Life sciences study design

All studies must disclose on these points even when the disclosure is negative.

| | |
|---|---|
| Sample size | The sample sizes were sufficient to observe statistically significant differences among various treatments or genotypes. The details about samples used in the study are reported in Figure legends. |
| Data exclusions | No data were excluded from this study. |
| Replication | Experiments were perfomed at least two times with similar results. |
| Randomization | Samples were grown on the same condition and randomly allocated in the growth chamber. Experimental plant materials were collected randomly without any bias. |
| Blinding | We did not apply blinding because the process of sample collection and analysis was carried out by the same person. Data were collected for each genotype or treatment. Plant samples were analyzed using objective, standardized protocols that include appropriate controls. |

# Reporting for specific materials, systems and methods

We require information from authors about some types of materials, experimental systems and methods used in many studies. Here, indicate whether each material, system or method listed is relevant to your study. If you are not sure if a list item applies to your research, read the appropriate section before selecting a response.

## Materials & experimental systems

| n/a | Involved in the study | |
|---|---|---|
| ☐ | ☒ | Antibodies |
| ☒ | ☐ | Eukaryotic cell lines |
| ☒ | ☐ | Palaeontology and archaeology |
| ☒ | ☐ | Animals and other organisms |
| ☒ | ☐ | Clinical data |
| ☒ | ☐ | Dual use research of concern |
| ☐ | ☒ | Plants |

## Methods

| n/a | Involved in the study | |
|---|---|---|
| ☒ | ☐ | ChIP-seq |
| ☒ | ☐ | Flow cytometry |
| ☒ | ☐ | MRI-based neuroimaging |

# Antibodies

| | |
|---|---|
| Antibodies used | Mouse anti-His-Tag mAb HRP-conjugated (1:5000 dilution, MBL, D291-7, clone: OGHis) |
| Validation | The anti-His-Tag antibody is verified for specificity on the developers website (https://ruo.mbl.co.jp/bio/e/dtl/A/?pcd=D291-7) |

# Dual use research of concern

Policy information about dual use research of concern

## Hazards

Could the accidental, deliberate or reckless misuse of agents or technologies generated in the work, or the application of information presented in the manuscript, pose a threat to:

| No | Yes | |
|---|---|---|
| ☒ | ☐ | Public health |
| ☒ | ☐ | National security |
| ☒ | ☐ | Crops and/or livestock |
| ☒ | ☐ | Ecosystems |
| ☒ | ☐ | Any other significant area |

## Experiments of concern

Does the work involve any of these experiments of concern:

| No | Yes | |
|---|---|---|
| ☒ | ☐ | Demonstrate how to render a vaccine ineffective |
| ☒ | ☐ | Confer resistance to therapeutically useful antibiotics or antiviral agents |
| ☒ | ☐ | Enhance the virulence of a pathogen or render a nonpathogen virulent |
| ☒ | ☐ | Increase transmissibility of a pathogen |
| ☒ | ☐ | Alter the host range of a pathogen |
| ☒ | ☐ | Enable evasion of diagnostic/detection modalities |
| ☒ | ☐ | Enable the weaponization of a biological agent or toxin |
| ☒ | ☐ | Any other potentially harmful combination of experiments and agents |
